# High fever and multi-nodular lung consolidations after whole lung lavage in a patient with pulmonary alveolar proteinosis

**DOI:** 10.1186/s40064-015-0849-2

**Published:** 2015-02-05

**Authors:** Shufang Zhang, Yesong Wang, Zhihao Xu, Liren Ding, Liuhong Wang, Libin Li, Gensheng Zhang

**Affiliations:** Department of Cardiology, Binjiang Branch, Second Affiliated Hospital, Zhejiang University School of Medicine, Hangzhou, Zhejiang 310009 China; Department of Critical Care Medicine, Second Affiliated Hospital, Zhejiang University School of Medicine, Hangzhou, Zhejiang 310009 China; Department of Respiratory Medicine, Fourth Affiliated Hospital, Zhejiang University School of Medicine, Yiwu, Zhejiang 322000 China; Department of Respiratory and Critical Care Medicine, Second Affiliated Hospital, Zhejiang University School of Medicine, Hangzhou, Zhejiang 310009 China; Department of Radiology, Second Affiliated Hospital, Zhejiang University School of Medicine, Hangzhou, Zhejiang 310009 China

**Keywords:** Pulmonary alveolar proteinosis, High fever, Multi-nodular lung consolidations, Whole lung lavage, Complication

## Abstract

**Introduction:**

Whole lung lavage is the most effective method to treat pulmonary alveolar proteinosis (PAP), and most potential complications occur often during the lavage process, but few happen after lavage. Theoretically, pulmonary edema would be more common after whole lung lavage. However, no such case was reported in the literature.

**Case description:**

A 47-year-old Chinese male patient with PAP was referred to our hospital for whole lung lavage treatment. Although the clinical manifestations of PAP were improved, high fever was happened and multi-nodular consolidations in chest CT scan were occurred after whole lung lavage. Secondary lung infection was suspected, but the patient was not treated with antibiotics immediately. After therapies like liquid limitation, glucocorticoid administration and diuretic treatment, the patient was improved gradually. Namely, newly nodular consolidations were almost completely absorbed in three days, along with the complete recovery of body temperature and associated inflammatory biomarkers. The diagnosis of secondary infection was excluded, and the final diagnosis of lavage fluid-induced pulmonary edema was confirmed.

**Discussion and evaluation:**

No such case has been reported that lavage fluid-induced pulmonary edema is manifested by high fever and multi-consolidations in chest CT scan, which is similar to the secondary infection.

**Conclusions:**

For the first time, we described a rare complication of lavage fluid-induced pulmonary edema after whole lung lavage. As the obvious differences in treatments, it is very important for physicians to differentiate it from secondary infection.

## Background

Pulmonary alveolar proteinosis (PAP) was first described in 1958 and classified idiopathic, secondary and congenital types (Seymour and Presneill 2002). Whole lung lavage is the most effective method, which is well tolerant and relatively safe (Michaud et al. 2009). Most potential complications for whole lung lavage occur often during the lavage process such as intraoperative refractory, low oxygen saturations, pneumothorax and hydropneumothorax (Michaud et al. 2009). In contrast, most patients recover well and few complications happen after lavage. Theoretically, pulmonary edema would be more common after whole lung lavage. However, no such case was reported in the literature. To our knowledge, we report for the first time here that lavage fluid-induced pulmonary edema serves as a rare complication of PAP after bilateral whole lung lavage, which is manifested by high fever and multi-nodular lung consolidations. In addition, this case was approved by the ethics committee of Second Affiliated Hospital, Zhejiang University School of Medicine.

## Case description

A 47-year-old male patient with increasing breathlessness and dry cough for a 1-year history was reported here. He has received a diagnosis of PAP in a local hospital, confirmed by radiological findings of bilateral patchy reticular opacities in both lung fields in chest computed tomography (CT) scan and pathological features of alveolar filling with amorphous materials with a positive stain of periodic acid-schiff by transbronchial lung biopsy. He didn’t take any medicine like steroid before.

**Figure 1:**
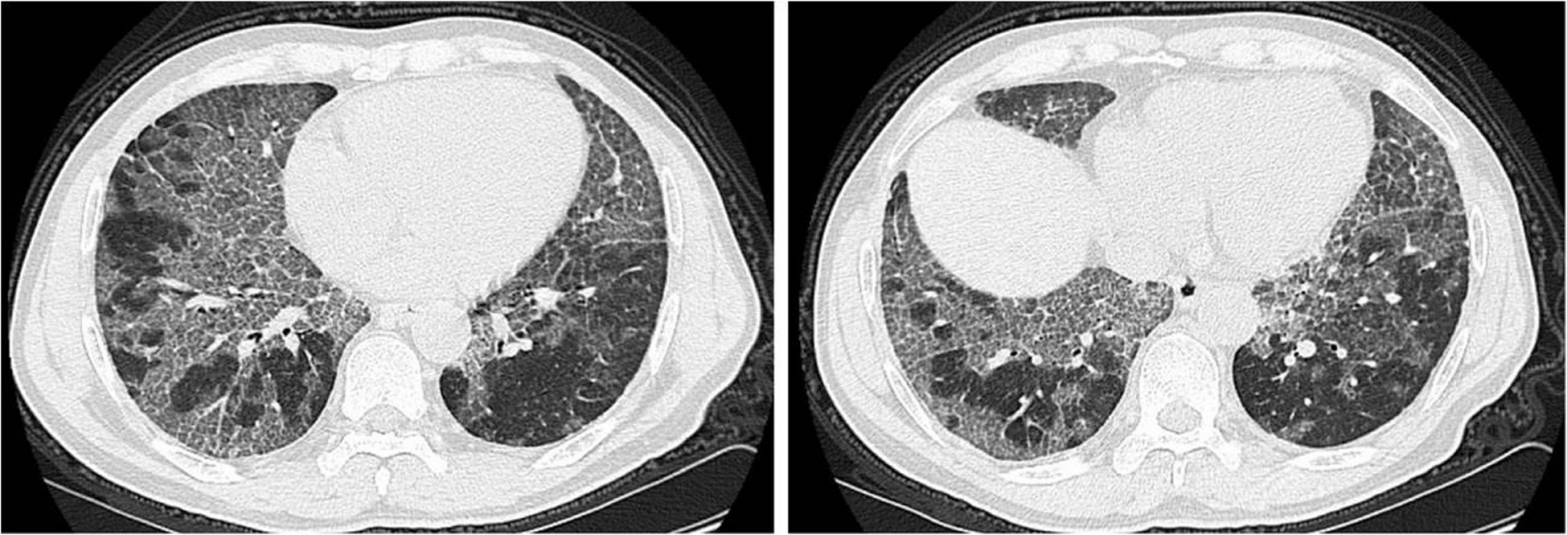
**Chest CT image before whole lung lavage showing a bilateral ground-glass opacity with interlobular septal thickening with a typical crazy paving pattern.**

The patient was referred to our hospital for treatment. He was tachypnoeic and tachycardic with fine crackles in both lungs. Arterial blood gas after oxygen supply (5 liters/min) showed as follows: pH 7.394, PCO_2_ 37.6 mmHg, PO_2_ 71.6 mmHg, and SaO_2_ 93.8%. Before lung lavage, chest CT scan showed a bilateral ground-glass opacity with interlobular septal thickening, characterizing by a crazy paving pattern (Figure 1). Bilateral whole lung lavage was performed on the third day after admission as described by Silva et al. (Silva et al. 2014). After general anesthesia and single-lung ventilation, the patient received lavage first on the left side with 4 liters of saline at 37°C and then on the right side with 6 liters of saline. The lavage fluid was initially turbid with sediment but gradually cleared. A sputum sample (approximately 10 mL) from right site was obtained from the second lavage and sent to the laboratory for culture. The total recovery rate of lavage fluid was 90%. Namely, the residual amount of liquid was 0.3 liters in the left site and 0.7 liters in the right site. After lung lavage, the patient was transferred to intensive care unite (ICU) for further treatment.

**Figure 2:**
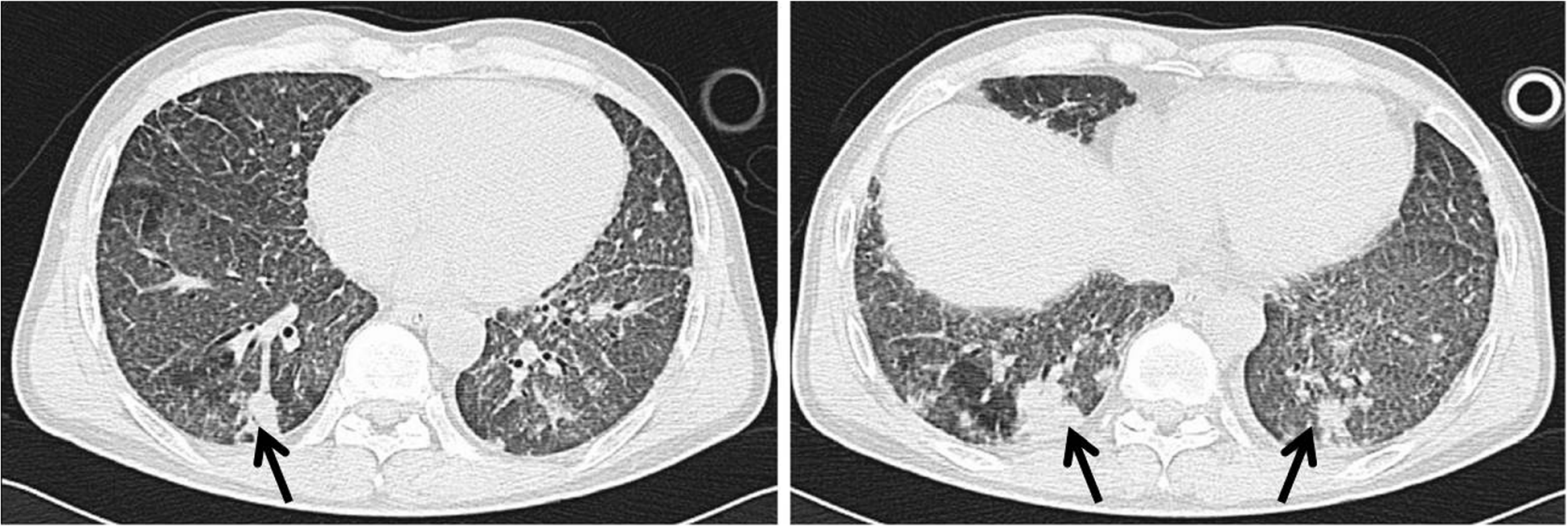
**Chest CT image performed one day after the whole lung lavage showing newly multi-nodular consolidations (black arrow) and patchy opacities in both posterior segments of lower lobes.**

**Figure 3:**
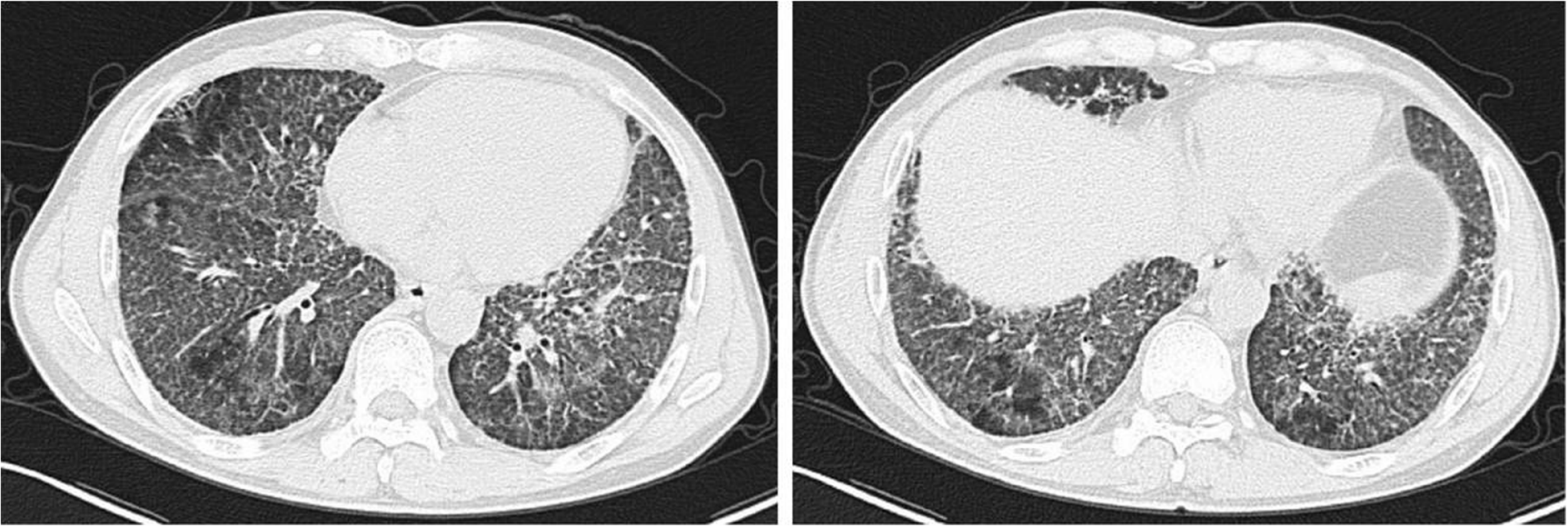
**Chest CT image conducted three days after the high fever showing almost complete absorption of the multipe nodular lung consolidations.**

The patient was extubated on the second day in ICU, but high fever was occurred. The body temperature was between 39.0 and 40.0°C. Blood routine test, blood and sputum cultures, and chest CT scan were performed rapidly. The white blood cell count was normal with a high percentage (95.2%) of neutrophils. In addition, newly multi-nodular consolidations occurred in both posterior segments of lower lobes in chest CT scan (Figure 2). A possible diagnosis of secondary lung infection cannot be excluded completely, but the patient was not treated by broad-spectrum antibiotics immediately and observed closely for vital signs. At the same time, treatments like liquid limitation, glucocorticoid administration and diuretic treatment were continued. The patient’s condition was improved gradually. The body temperature was returned to normal level three days later. Importantly, multi-nodular lung consolidations were absorbed almost completely in chest CT scan (Figure 3). There was no bacterial/fungi growth from samples of blood, sputum and lavage fluid. The value of C-reactive protein (CRP) was dropped from high level of 223.9 mg/L to 10.5 mg/L (normal range, < 10 mg/L), and procalcitonin (PCT) was also recovered from 0.71 ng/mL to normal level of 0.10 ng/mL (normal range, < 0.5 ng/mL). The secondary infection was excluded, and the final diagnosis of lavage-induced pulmonary edema was confirmed. The patient was asymptomatic at discharge two weeks later with improved SpO_2_ (93.0 ~ 95.0% in room air), and remained well within six months of follow-up after discharge.

## Discussion

A puzzle in this case was high body temperature after whole lung lavage. Persistent high fever, high percentage of neutrophils in blood test, and multiple lung consolidations in bilateral lower fields made it hard to exclude the possibility of secondary bacterial/ fungal infection. In addition, patients with PAP are susceptible to secondary/opportunistic infections due to impaired functions of macrophages and neutrophil (Punatar et al. 2012). However, the initial diagnosis of secondary pulmonary infection was not supported eventually evidenced by as follows: (i) Subsequent culture results from samples of lavage fluid, blood or sputum were negative. (ii) The clinical manifestations were improved after therapies like liquid limitation, glucocorticoid administration and diuretic treatment. In addition, pulmonary edema-induced consolidations are often absorbed completely 72 hour later after effective treatment (Gluecker et al. 1999), which is consistent with our case. (iii) Compared with CRP, PCT is a relatively specifical marker of infection such as respiratory infection or sepsis. A relatively high level of PCT above 2.0 ng/mL has a highly positive predictive value for sepsis or severe sepsis (BalcI et al. 2003). In the present patient, serum PCT was only slightly elevated (0.71 ng/mL) at the time point of high fever. Since other conditions like operative stress may also result in a transitory increase in PCT level (Meisner et al. 1998), the relatively elevated PCT in the present case might represent an acute stress situation of whole lung lavage. Therefore, the diagnosis of lavage fluid-induced pulmonary edema was finally established. Since the high fever can not be explained by secondary lung infection after lung lavage, it is possible that the secondary sterile inflammation and subsequent large absorption of lavage fluid may ascribe to the high body temperature in the current patient.

Not like cardiogenic pulmonary edema, the manifestations of lavage-induced pulmonary edema in CT scan in the current patient display asymmery focal consolidations in posterior segments of bilateral lower fields. Compared to other segments, liquids are relatively hard to be drained completely at basal segments of lower lobes after whole lung lavage. Similar to the current case, focal consolidations in CT scan are also observed in near-drowning patients as a significant feature of pulmonary edema (Gregorakos et al. 2009). Mechanically, residual lavage fluids in the lung lead to local hypoxia, which causes subsequent permeability edema resulting from the releases of inflammatory cytokines and the dmages of capillary and alveolus in corresponding lung fields as observed in Stage 3 of near drowning pulmonary edema (Gluecker et al. 1999). For this case, the rapid improvements in clinical and radiological manifestations might be associated with the fast absorption of residual lavaged fluids from the lung into circulation after liquid limitation and diuretic treatment.

As described in previous studies (Silva et al. 2014; Luisetti et al. 2010; Rebelo et al. 2012), a manual percussion of the alveoli with several cycles of manual ventilation using a continuous positive airway pressure (CPAP) valve with 5 ~ 10 mmHg pressure limit may enhance the removal of the accumulated material. However, we did not perform a percussion of the alveoli in the current case, which might ascribe to the formation of pulmonary edema.

Although the final diagnosis was lavage fluid-induced pulmonary edema (Figure 2), it should be differentiated from other clinical conditions such as organizing pneumonia, alveolar damage, and mechanical oedema. The multi-nodular lung consolidations were occurred just one day after lung lavage and almost completely absorbed in three days later. In addition, the patient had no relapse during the period of hospitalization and the six months of follow-up after discharge; However, organising pneumonia has a subacute presentation between 1 and 12 weeks, remains some residual changes like fibrotic non-specific interstitial pneumonia in CT scan after effective treatments, and is easy to relapse (Beardsley and Rassl 2013). The features including a rapid onset, a quickly complete absorption of multi-nodular lung consolidations, and no relapse in the current case suggested that it was not likely organising pneumonia. As the saline was instilled into the lung under gravitational effect from a height not exceeding 40 centimeters above mid-axillar line according to the procedure described previously (Silva et al. 2014), it might not cause the alveolar damage or mechanical oedema by the lavage itself. In addition, ventilator associated lung injury occurs often in the conditions of high inspiratory transpulmonary pressure (barotrauma), alveolar overdistention (volutrauma), repetitive opening and closing of collapsed alveoli (atelectrauma) and biotrauma (Sutherasan et al. 2014). However, the parameters of mechanical ventilator in the case were at a low level with a fraction of inspiratory oxygenof 40%, a pressure support of 12 cmH_2_O, and a tidal volume of 8 ml/kg. Thus, it might be impossible to cause ventilator associated lung injury in the case.

## Conclusion

We presented a rare complication of lavage fluid-induced pulmonary edema after whole lung lavage in a patient of PAP, which was manifested by high fever and multi-nodular lung consolidations in CT scan, and suspected as secondary infection initially. As the obvious differences in treatments, it is very important for physicians to differentiate lavage fluid-induced pulmonary edema from secondary infection in patients with PAP after whole lung lavage.

## Availability and requirements

The article type is Case study.

## Abbreviations

PAP: Pulmonary alveolar proteinosis
CT: Computed tomography
ICU: Intensive care unit
CRP: C-reactive protein
PCT: Procalcitonin
